# Effects of a Multi-component, Resistance-Based Exercise Program Combined with Additional Lean Red Meat on Health-Related Quality of Life in Older Adults: Secondary Analysis of a 6-Month Randomized Controlled Trial

**DOI:** 10.1007/s12603-023-1915-1

**Published:** 2023-04-28

**Authors:** M.B. Formica, J. Gianoudis, C.A. Nowson, S.L. O'Connell, C. Milte, K.A. Ellis, Robin M. Daly

**Affiliations:** 1 Institute for Physical Activity and Nutrition (IPAN), School of Exercise and Nutrition Sciences, Deakin University, 221 Burwood Highway, Burwood, 3215, Melbourne, VIC, Australia; 2 Neurodegeneration Division, The Florey Institute, Academic Unit for Psychiatry of Old Age, Department of Psychiatry, The University of Melbourne, Melbourne, Australia

**Keywords:** Lean red meat, dietary protein, exercise, quality of life, older adults

## Abstract

**Objectives:**

To assess whether consumption of lean red meat on three exercise training days per week can promote greater improvements than exercise alone in health-related quality of life (HR-QoL) in community-dwelling older adults.

**Design:**

This study is a secondary analysis from a 6 month, two-arm, parallel randomized controlled trial conducted in 2014 and 2015.

**Setting:**

Community-dwelling older adults living in metropolitan Melbourne, Australia.

**Participants:**

One hundred and fifty-four men and women aged ≥65 years.

**Intervention:**

All participants were enrolled in a multi-component, resistance-based exercise program (3 d/week) and randomly allocated to either a group asked to consume lean red meat (2x80g cooked servings/day) on each of the three training days (Ex+Meat, n=77) or a control group asked to consume one serving of carbohydrates (1/2 cup rice/pasta or 1 medium potato; Ex+C, n=77).

**Measurements:**

HR-QoL was assessed using the Short-Form (SF)-36 health survey.

**Results:**

Overall 62% of the participants were female, the mean age was 70.7 years (range 65 to 84 years), approximately 67% of participants were classified as either overweight or obese, and the average number of chronic conditions was two. A total of 145 participants (94%) completed the study. Mean baseline HR-QoL scores were comparable to the mean for the Australian population [Global HR-QoL (mean ± SD): Ex+Meat, 49.99 ± 6.57; Ex+C, 50.49 ± 5.27]. General Linear Mixed Models examining within and between group changes over time revealed that after 6 months, there were no within-group changes in either Ex+Meat or Ex+C nor any between-group differences for any measure of HR-QoL, with the exception that the mental health subscale improved in Ex+C versus Ex+Meat [net difference for change, −2.32 (95% CI), −4.73, 0.09, P=0.048] after adjusting for relevant covariates and the physical function subscale improved in Ex+Meat relative to baseline [mean change (95% CI), 1.88 (0.37, 3.39), P=0.011].

**Conclusion:**

A multi-component resistance-based training program performed with and without the provision of lean red meat in line with current Australian dietary guidelines on each of the three training days, did not improve HR-QoL in healthy community-dwelling older adults.

## Introduction

Increased levels of chronic disease and illness associated with a global aging population and increasing life expectancy can significantly impact well-being, levels of independence and health-related quality of life (HR-QoL) (1). Although there are many factors which can impact HR-QoL, including environmental and situational factors (e.g. financial status, changes in living conditions, death of spouse/family member), there is emerging evidence that age- and disease-related losses in skeletal muscle mass, strength and function are associated with reduced HR-QoL (2, 3, 4, 5, 6). This may be related, in part, to a loss of independence, which can lead to declines in other psychological constructs including self-esteem and self-efficacy and an onset of depressive symptoms. Therefore, strategies which target the prevention of age-related losses in muscle in older adults may also play a key role in ameliorating age and/or disease-related deteriorations in HR-QoL.

Current clinical and practice guidelines recommend a combination of adequate dietary protein and progressive resistance training (PRT) to optimise muscle mass, strength and function in older adults (7, 8, 9), but whether the combination can result in greater improvements in HR-QoL remains uncertain. There is some promising evidence from several randomised controlled trials (RCTs) in pre-frail and sarcopenic older adults to support an additive benefit of additional protein with exercise on HR-QoL (10, 11). For instance, a 12-week RCT conducted in 130 sarcopenic older adults reported improvements in both the physical and mental component scores following a moderate intensity multi-component exercise program combined with an amino acid/whey-protein (22 g/d) and vitamin D (100 IU/d) enriched supplement, compared to exercise alone (11). Further to this, in a secondary analysis of a previous 4-month RCT in healthy older women that examined the effects of twice-weekly PRT combined with consumption of ~160g (cooked) of lean red meat on six days a week as an approach to increase daily dietary protein intake, we reported greater improvements in the PRT plus lean red meat group compared to PRT alone in overall HR-QoL and the physical component score (PCS) of the Short Form 36 health survey version 2 (SF36-v2) (12). Furthermore, we demonstrated that PRT plus lean red meat induced greater increases in lean tissue mass and lower limb muscle strength, with the latter positively associated with changes in overall HR-QoL (12). However, the dose of red meat used in this study was well above the current Australian dietary guidelines which recommend that adults consume a maximum of 455g or 3–4 serves of trimmed red meat per week (13). Thus, it is unknown whether consuming lean red meat in line with the Australian dietary guidelines, when combined with resistance-based exercise, can similarly improve HR-QoL in older adults.

This study is a pre-specified secondary analysis from a 6-month randomized controlled trial for which we have previously reported that twice-daily consumption of lean red meat on each of the three exercise training days per week, aimed at increasing protein intake to approximately 1.3 g/kg body weight/day, did not augment exercise-induced improvements in total body or leg lean mass, leg muscle strength, fat mass or physical function (sit-to-stand and four-square step test), compared to exercise training alone in healthy community-dwelling adults, but did result in significantly greater improvements in arm lean mass and gait speed (14). In addition, per protocol analysis of participants most adherent to the intervention revealed that exercise plus lean red meat led to significantly greater gains in appendicular lean mass compared to exercise alone (14). Therefore, the aim of this study was to determine whether consumption of lean red meat combined with resistance-based exercise on three days per week, can promote greater improvements than exercise alone on HR-QoL in community-dwelling older men and women.

## Subjects and methods

### Study Design

A detailed description of the study methods has been previously reported (15). Briefly, 154 community-dwelling healthy older adults were prescribed a multi-component exercise program to be performed three days per week for 6 months, and were randomized to receive either: 1) lean red meat to be consumed as two, 80g (cooked) servings on each of the three training days (Ex+Meat, n=77); or 2) half a cup of cooked carbohydrates (i.e. rice, pasta or potato) to be consumed as part of the usual diet on each of the training days (Ex+C, n=77). The term ‘healthy' in this study refers to older adults without significant chronic health conditions which limit physical movement, cognitive status or mood (further details are provided in the exclusion criteria below). An independent researcher (not involved in the study) randomized participants at the level of the individual in blocks of four, stratified by gender, using a computer-generated random number sequence. To avoid contamination, couples (n=17) were randomized to the same condition. Participants and research staff were not blinded to the group allocation, although those analysing the data were blinded. The Deakin University Human Research Ethics Committee (HREC 2013-166) approved the study, and it was registered with the Australian and New Zealand Clinical Trials Registry (ACTRN12613001153707) with the study formatted in accordance with CONSORT guidelines (16).

### Participants

Men and women aged 65 years and over residing in the community in metropolitan Melbourne and surrounding suburbs were recruited into this trial via several approaches, including newspaper advertisements, word of mouth, flyers on community notice boards and presentations at local community centres. Persons who expressed interest were screened using a four-step process (15). Participants were initially excluded via telephone screening based on the following criteria: aged <65 years; current or prior participation in structured resistance training (>once per week) and/or moderate intensity physical activity (≥150 minutes per week) over the last three months; BMI >40 kg/m^2^; acute or terminal illness, including current (treated or untreated) cancer or surgery/cessation of chemotherapy/radiotherapy <12 months ago; history of a recent low trauma fracture that would limit involvement in the exercise program; diabetes treated with insulin; chronic liver disease, coeliac or inflammatory bowel disease, including ulcerative colitis or Crohn's disease; use of oral corticosteroids (past six months); or not able to commit to the study requirements. If participants were currently taking vitamin D supplements, they were asked to substitute these with the vitamin D capsules provided to them as part of the study. Eligible participants were then asked to complete the Short Portable Mental Status Questionnaire (SPMSQ) and the Geriatric Depression Scale (GDS) to confirm that they were cognitively healthy and exhibited no signs of depression, respectively. A score of greater than two on the SPMSQ and/or a score of greater than six on the GDS led to exclusion from the study. Remaining eligible participants were then assessed by their local doctor for any contraindications to exercise, based upon the American College of Sports Medicine (ACSM) guidelines (17). Finally, participants were asked to provide a fasted, morning blood sample to confirm that their estimated Glomerular Filtration Rate (eGFR) was >45 mL/min/1.73m^2^.

Recruitment and testing of participants was staggered over two years (cohort 1, 2014 and cohort 2, 2015) to make implementation of the intervention more manageable. The intervention began in April each year with 68 participants recruited in the first year and 86 in the second year. There were no significant differences in any of the baseline demographic or HR-QoL characteristics between the participants in cohort 1 or 2 (data not shown).

### Exercise Intervention

Specific information about the exercise intervention has been reported previously (15). Briefly, all participants were prescribed an individually-tailored, progressive multi-component resistance exercise program comprising three sets of 12–15 repetitions progressing to 8–12 repetitions at moderate to high intensity (5 to 8 of 10-point Borg RPE scale), challenging balance and mobility activities (at least two exercises) and aerobic exercise (15–20 minutes) at moderate intensity (rating 5 to 8 on 10-point Borg Rating of Perceived Exertion (RPE) scale), performed on three non-consecutive days a week for 6 months. All training was conducted in small groups (2–10 participants) at one of 15 local community health and fitness centres in metropolitan Melbourne and surrounding areas, at no cost to the participants. Each exercise session was approximately 60–75 minutes in duration and supervised by qualified exercise trainers. Where possible, exercise sessions were conducted mid-morning or late afternoon to facilitate the consumption of lean red meat or carbohydrates at lunch or dinner and as close to the training time as possible (ideally within two hours).

### Dietary Intervention

#### Lean Red Meat

Participants randomized to the Ex+Meat group were supplied with lean red meat (∼220 g/day, raw weight) that included varied cuts of beef, lamb and veal, trimmed of visible fat, to be consumed on each of their three training days, across two meals (e.g., lunch and dinner; ∼80 g cooked serving/meal). This equates to ∼160 g of cooked meat per day (∼45 g protein), or approximately 1.3 g/kg/d. Participants were instructed to maintain their normal diet on non-training days. If participants could not consume the meat across two meals, they were advised to consume both portions after their training session. Compliance was monitored via a daily calendar which required participants to record all meat consumed each day. This was returned to the researchers each month.

#### Control group

Participants randomized to the carbohydrate (control) group were instructed to consume their usual diet on both training and non-training days as well as a minimum of half a cup of cooked rice or pasta or one medium sized potato (∼25–35 g of carbohydrates) on each of the training days. Participants were provided with packs of pasta and rice by the research team. On the non-training days, participants were advised to prioritise larger servings of carbohydrates (i.e., breads, cereals, rice, pasta and vegetables) and smaller amounts of protein foods (e.g., meat, fish and chicken). The inclusion of carbohydrate foods was designed to keep the participants' dietary protein intake less than 1.1 g/kg per day and to ensure that both groups received the same level of researcher attention, rather than to achieve an isoenergetic diet. A checklist of carbohydrate-rich meals (pasta, potato and rice) was completed daily to measure compliance.

### Vitamin D supplementation

To optimise vitamin D status (serum 25-hydroxyvitamin D concentration ≥50–60 nmol/L), all participants were prescribed one 1000 IU vitamin D3 capsule (Blackmores, Australia) per day. Adherence was monitored by counting returned capsules at the end of the study.

### Health-related quality of life

Health-related Quality of Life (HR-QoL) was assessed at baseline and 6 months via self-report using the Short Form health survey 36 version 2 (SF36-v2) (18). This is a 36-item questionnaire that consists of eight subscales [physical functioning (10 items), bodily pain (2 items), general health (5 items), vitality (4 items), social functioning (2 items), mental health (5 items), role-emotional (3 items) and role-physical (4 items)] (19).

From the eight subscales, two summary scores were created: 1) physical component summary scale (PCS) and 2) mental component summary scale (MCS). The mean of all the eight health subscales was used to calculate an overall HR-QoL score ranging from 0 to 100, with higher scores representing a better rating of HR-QoL. Previously published Australian norm-based scores (20) were used when reporting results so that a change in scores could be assessed meaningfully. These norm-based weights give each domain score a mean of 50 and a standard deviation (SD) of 10.

### Anthropometry

Height (to nearest 0.5 cm) was measured using a stadiometer (Holtain, UK). Weight (in kg) was measured with participants dressed in gowns and underwear using calibrated electronic digital scales (Seca, model 708). Body mass index (BMI) was calculated as weight (in kg) divided by height (in meters) squared. All anthropometry measures were taken at baseline and 6 months.

### Demographic and health/medical history

Demographics [age, sex, ethnicity (Caucasian or non-Caucasian), educational history (did not complete high school, completed high school, technical/ trade certificate, University or tertiary qualification)], living arrangement (living alone or with another adult/children) number of chronic health conditions (current and past), smoking status (non-smoker or ex/current smoker) and current use of prescription medications were collected via questionnaire. Participants were also required to report any changes in use, or any new medications commenced throughout the duration of the study. Habitual physical activity (moderate to vigorous, kilojoules per week) was assessed at baseline and 6 months using the Community Healthy Activities Model Program for Seniors (CHAMPS) self-report questionnaire, which is validated for use in older adults (21).

### Depression and anxiety

The Hospital Anxiety and Depression Scale (HADS) was used to assess symptoms of depression and anxiety at baseline and 6 months. HADS is a 14-item self-report questionnaire divided into two subscales, with seven questions assessing cognitive and emotional aspects of anxiety, and the other seven questions evaluating cognitive and emotional aspects of depression (22). Higher scores indicate a greater severity of anxiety and/or depression symptoms (22).

### Statistical Analysis

As reported previously (15), the number of participants required for this study was based on the expected difference between the groups for the primary outcomes of total body lean mass. As previously recommended (23), no post-hoc power calculations were performed for the secondary analysis of HR-QoL scores.

Statistical analyses were conducted on an intention-to-treat (ITT) basis using STATA statistical software version 17.0 (STATA, College Station, TX, USA). Data from participants who withdrew from the exercise program or intervention were included if end-point measures were obtained. A pre-specified per protocol analysis was also performed for participants with at least 66% adherence to the exercise and 80% adherence to the dietary interventions. General Linear Mixed Models, with participant as the random effects, time as a repeated measure, and group-by-time interactions as the fixed effects, were used to analyse the data. The results were analysed unadjusted and adjusting for the following covariates: age, sex, number of chronic diseases, change in physical activity, depression/anxiety and living arrangement. Normality and homogeneity of variance of the residuals were checked using quantile-quantile plots and scatterplots, respectively. Within-group changes in the outcome measures were expressed as either the absolute or percentage change from baseline. Between-group differences were calculated by subtracting the within-group changes from baseline for Ex+Meat from the within-group changes for Ex+C after 6 months. All data is presented as means with standard deviations (SD) or 95% confidence intervals (CI) unless specified otherwise. P<0.05 was used to determine statistical significance.

## Results

### Baseline characteristics

**Figure 1:**
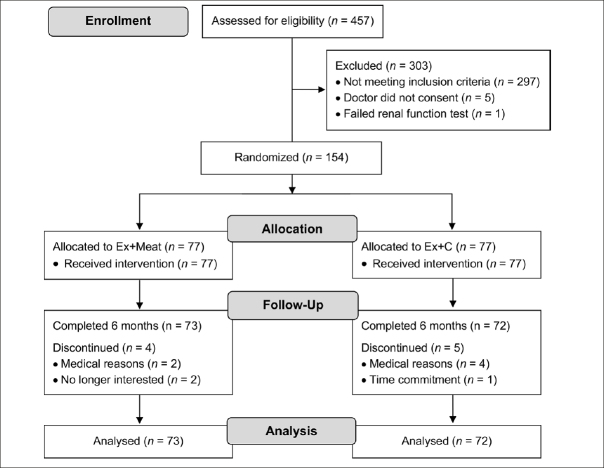
Flowchart of participants through the study Ex+Meat, exercise plus lean red meat; Ex+C, exercise plus carbohydrate control.

One hundred and fifty-four healthy older adults were randomised to one of the two groups (Ex+Meat, n=77 or Ex+C, n=77) (Figure 1). Approximately two-thirds (62%) of the participants were female and the mean age was 70.7 years (range 65 to 84 years). Fifty one percent of participants were currently married, 56% were either separated or divorced, 31% were widowed and 37% were living alone. The mean BMI was 27.9 kg/m^2^ with 35.1% of the participants classified as overweight and 31.8% as obese (Table 1). The average number of chronic health conditions was approximately two. More than 80% of participants in each group were taking prescribed medications, with 60–72% taking two or more medications.

**Table 1:** Baseline characteristics of the multi-component exercise and lean red meat (Ex+Meat) and control (carbohydrate) multi-component exercise (Ex+C) group

**Characteristic**	**Ex+Meat**	**Ex+C**
N	77	77
Women, n (%)	48 (62)	48 (62)
Age (years)	71.2 ± 4.0	70.3 ± 4.3
BMI (kg/m^2^)	27.8 ± 5.1	27.9 ± 5.5
Normal weight (BMI 18.5–24.9), n (%)	23 (30)	28 (36)
Overweight (BMI 25–29.9), n (%)	32 (42)	22 (29)
Obese (BMI ≥ 30), n (%)	22 (29)	27 (35)
Educational history		
Did not complete high school	14 (18)	17 (22)
Completed high school	14 (18)	12 (16)
Technical / trade certificate	15 (20)	10 (13)
University or tertiary level	34 (44)	38 (49)
Ethnicity*, n (%)		
Caucasian	66 (86%)	63 (82%)
Non-caucasian	11 (14%)	14 (18%)
Currently married, n (%)	40 (52%)	38 (49%)
Living alone, n (%)	26 (34%)	31 (40%)
Ex-smoker or current smoker, n (%)	30 (39)	26 (34)
Moderate-vigorous PA, kJ per week	7897 ± 7555	8017 ± 7547
Taking prescribed medication, n (%)	68 (88)	65 (84)
One medication	12 (16)	19 (25)
Two or more medications	55 (72)	46 (60)
Number of chronic health conditions	2.1 ± 1.7	2.2 ± 1.8
Depression/Anxiety symptoms#, n (%)	2 (3)	1 (1)

Values represent number and percentage or means ± SDs. PA, physical activity; CVD, cardiovascular disease. *Caucasians included those born in Australia, Northern Europe, Canada, the United States, and New Zealand; Non-Caucasians included those born in Southern Europe, Asia, the Middle East, India and Sri Lanka, Pacific Islands, Africa, South and Central America, Aboriginal Australians, and Torres Strait Islanders. # Depression/anxiety symptoms classified as scores 8 or above on the Hospital Anxiety and Depression Scale.

### Study Attrition and Adverse Events

Nine participants (Ex+Meat, n=4; Ex+C, n=5) did not complete the 6-months for consistency testing, citing medical reasons (Ex+Meat, n=2; Ex+C, n=4), time commitment (Ex+C, n=1) or loss of interest in the study (Ex+Meat, n=2) as reasons for withdrawing. A comparison between those who withdrew from the study and those who remained revealed no differences regarding any outcomes assessed at baseline (data not shown). No serious adverse events were reported throughout the intervention, although 13 participants had to cease training for a short period (range 1 to 4 sessions) due to exercise-related injury or pain. Overall, three participants withdrew from the exercise due to either aggravation of a pre-existing injury or pain (n=2) or a new complaint unrelated to the intervention (n=1). However, these participants returned for the follow-up assessments.

### Program Adherence and Compliance

As previously reported (14), the mean adherence to the exercise training for all participants was ~78% and did not differ between the groups (Ex+Meat: 77.9% ± 19.2 and Ex+C: 78.6% ± 20.4). Mean ± SD dietary compliance was 87.0% ± 19.8 in the Ex+Meat group and 91.3% ± 22.6 in the Ex+C group. Compliance with the vitamin D supplementation was high and did not differ between the groups (Ex+Meat: 94.6% ± 10.1; Ex+C: 92.6% ± 11.3).

### Effects of the intervention on HR-QoL

Mean baseline Global HR-QoL scores and subscale scores for both groups were consistent with previously reported Australian norms (Table 2). There were no within-group changes or between group differences for the change (unadjusted and adjusted for covariates) for any measure of HR-QoL after 6 months (Table 2), except for the physical function subscale which improved by 1.88 units (95% CI, 0.37, 3.39) in the Ex+Meat group relative to baseline (P=0.011). In addition, after adjusting for covariates there was a significant difference (net benefit) in favour of the Ex+C for the change in the mental health subscale [net difference (95% CI), −2.32 (−4.73, 0.09), P=0.048].

**Table 2:** Mean baseline scores for health-related Quality of Life for the exercise plus lean red meat (Ex+Meat) and exercise plus control carbohydrate (Ex+C) group, and the mean within-group changes and mean net differences between the groups for the change after 6 months

	**Baseline Values and Within-Group Changes**					
	**Ex+Meat**		**Ex+C**		**Intervention Effects**	
**P-value**	**Mean ± SD or (95% CI)**	**P-value**	**Mean ± SD or (95% CI)**	**P-value**	**Net Difference (95% CI)**	**P-value Model 1 | Model 2**
Physical Function						
Baseline	47.52 ± 7.41		48.23 ± 7.05			
Δ 6 months	1.88 (0.37, 3.39)	0.011	1.33 (−0.29, 2.96)	0.084	0.55 (−1.65, 2.75)	0.637 | 0.487
Role Physical						
Baseline	48.16 ± 8.42		50.45 ± 7.00			
Δ 6 months	0.99 (−1.07, 3.04)	0.293	−0.48 (−2.59, 1.62)	0.578	1.47 (−1.45, 4.39)	0.257 | 0.226
Bodily Pain						
Baseline	48.28 ± 10.97		47.69 ± 10.27			
Δ 6 months	−1.30 (−3.46, 0.85)	0.246	−0.38 (-−2.53, 1.77)	0.848	−0.92 (−3.94, 2.09)	0.497 | 0.468
General Health						
Baseline	51.10 ± 7.96		51.35 ± 6.92			
Δ 6 months	1.21 (−0.23, 2.64)	0.113	−0.02 (−1.26, 1.23)	0.983	1.22 (−0.66, 3.11)	0.235 | 0.203
Vitality						
Baseline	53.74 ± 8.61		53.35 ± 6.64			
Δ 6 months	−0.32 (−2.18, 1.55)	0.696	0.17 (−1.24, 1.58)	0.725	−0.48 (−2.80, 1.84)	0.599 | 0.628
Social Functioning						
Baseline	50.73 ± 8.85		52.33 ± 6.63			
Δ 6 months	−0.31 (−2.29, 1.67)	0.781	−1.87 (−3.79, 0.06)	0.093	1.56 (−1.18, 4.30)	0.311 | 0.327
Role-emotional						
Baseline	48.38 ± 9.77		49.80 ± 8.81			
Δ 6 months	1.70 (−0.63, 4.02)	0.129	−0.54 (−2.99, 1.91)	0.486	2.23 (−1.11, 5.58)	0.126 | 0.150
Mental Health						
Baseline	52.08 ± 8.30		50.74 ± 6.97			
Δ 6 months	−0.85 (−2.51, 0.82)	0.312	1.47 (−0.30, 3.24)	0.086	−2.32 (−4.73, 0.09)	0.053 | 0.048
Physical Component Score (PCS)						
Baseline	48.43 ± 8.28		49.34 ± 7.94			
Δ 6 months	0.82 (−0.77, 2.41)	0.292	0.17 (−1.55, 1.89)	0.795	0.65 (−1.67, 2.97)	0.594 | 0.493
Mental Component Score (MCS)						
Baseline	51.94 ± 9.13		51.96 ± 7.77			
Δ 6 months	−0.15 (−2.04, 1.74)	0.876	−0.07 (−1.89, 1.75)	0.720	−0.08 (−2.68, 2.52)	0.878 | 0.976
Global HR-QoL Score						
Baseline	50.00 ± 6.57		50.49 ± 5.27			
Δ 6 months	0.38 (−0.81, 1.56)	0.514	0.14 (−1.02, 1.30)	0.957	0.24 (−1.40, 1.88)	0.612 | 0.602

All baseline values are unadjusted means ± SD. All change values are unadjusted means (95% CI) and were calculated from the absolute difference from baseline. Mean net differences (95% CI) were calculated by subtracting the within-group changes for the Ex+Meat group from the within-group changes for the Ex+C group after 6 months. Number of participants by group at baseline and 6 months: Ex + Meat, n = 77 and n = 73; C + Ex, n = 77 and n = 72. P-values for model 1 are based on an unadjusted model while model 2 included age, sex, number of chronic diseases, change in physical activity, living arrangement and depression/anxiety as covariates. HR-QoL: health-related quality of life; PCS: physical component score; MCS: mental component score.

### Per-protocol analysis

The per-protocol analysis included 58 participants in Ex+Meat and 64 participants in Ex+C. As shown in Supplementary Table 1, there was a significant within-group improvement in the physical function subscale for Ex+Meat [2.37 (95% CI, 0.90, 3.84), P=0.001], but this was not significantly different from Ex+C (interaction, P=0.29). For the role-physical subscale there was also a significant improvement in Ex+Meat [2.40 (95% CI, 0.33, 4.47), P=0.02] but no significant change in Ex+C [−0.40 (95% CI, −2.67, 1.88), P=0.69]. This resulted in a trend for a significant group-by-time interaction after 6 months [mean difference, 2.80 (95% CI, −0.27, 5.86), P=0.07] which became significant in the adjusted model (P=0.048). Similarly, there was a trend for a greater improvement in the role-emotional subscale in Ex+Meat compared to Ex+C [net difference, 3.80 (95% CI, −0.14, 7.74), P=0.08], which was driven by a significant improvement in Ex+Meat [3.12 (95% CI, 0.60, 5.64), P=0.013] and no change in Ex+C [−0.68 (95% CI, −3.71, 2.35)]. Finally, for the mental health subscale, Ex+C had a significant 1.73 unit [(95% CI, −0.17, 3.62), P=0.05] increase relative to baseline.

## Discussion

The main finding from this secondary analysis of a 6-month RCT in community-dwelling older adults was that a multi-component, resistance-based exercise program, alone or in combination with lean red meat (two ∼80g servings cooked) consumed on each of the three training days, was not associated with any significant improvements in HR-QoL, except for a modest improvement in the physical function subscale in the Ex+Meat group and a modest net benefit in the mental health subscale in the Ex+C group in the fully adjusted model.

Previous meta-analyses and systematic reviews of intervention studies have reported that physical activity and/or exercise training can have a positive effect on HR-QoL, including both the physical and mental health domains in middle-aged and older adults (24, 25, 26). It has been suggested that this may be related to concurrent improvements in physical function and muscle strength, as well as increases in energy, freedom from pain and ability to undertake activities of daily living (12, 27, 28). In our study, the multi-component exercise program alone or in combination with the lean red meat did not result in any consistent improvements in overall HR-QoL or any of its subdomains, despite some significant overall exercise-related improvements in lean tissue mass, muscle strength and measures of physical function (14). In contrast to these findings, in our previous 4-month RCT in older women we found that PRT combined with lean red meat was associated with improvements in overall HR-QoL and PCS scores compared to PRT alone (12). While it is difficult to explain these contrasting findings given that the baseline physical and demographic characteristics of participants were similar between studies and participants in both studies exhibited exercise-related improvements in muscle strength and function, it may be related to other differences in the exercise program (PRT alone versus a multi-component training program), frequency of lean red meat prescribed (six versus three days per week) and/or the duration of the intervention (four vs six months). Furthermore, it is unlikely that dietary factors or habitual physical activity influenced our findings. As we have previously reported (14), the additional dietary protein (lean red meat) provided in our study was effective for increasing dietary protein intake (∼1.4 g/kg body weight per day) but there were no other observed dietary differences between the groups (except for iron and zinc). In addition, overall habitual physical activity levels outside of the exercise program did not differ between the groups throughout the study (14), but to ensure it did not influence our findings we included change in physical activity as a covariate in our fully adjusted model.

Given that HR-QoL has been shown to be influenced by many interrelated aspects of life related to illness, chronic conditions, disability and socioeconomic status (29, 30, 31), the lack of any marked improvements (or differences) in our study may be related to the fact that participants were relatively healthy, community-dwelling older adults. As we previously reported (14), this is highlighted by the finding that only a small proportion (15%) of our participants reported a history of CVD, and baseline HR-QoL scores were comparable to that of the Australian population (20). Although most participants did report having a one or more chronic health conditions, on average 60 to 72% of participants reported taking two or more medications, which implies these conditions were likely being appropriately managed. There is some evidence that exercise may be effective for improving measures of HR-QoL in older adults with various chronic conditions compared to healthy controls (25) and those with low HR-QoL scores at baseline (10).

Given that the prescribed intensity, frequency and duration of our multi-component exercise program was consistent with previous exercise studies that were included in meta-analyses and RCTs which have reported exercise-related improvements in HR-QoL in healthy and clinical populations (32, 33, 34, 35), another potential explanation for the lack of any exercise effects on HR-QoL in our study may relate to the timing of the follow-up assessment for HR-QoL. For instance, a meta-analysis of 32 studies found that acute/short-term training (programs <12 weeks in duration) were associated with significantly greater improvements in psychological well-being compared to programs >12 weeks in duration (36). Furthermore, another meta-analysis of 36 physical activity intervention studies reported that longer exercise duration was less beneficial for several measures of well-being (anxiety, depression and self-efficacy) in older adults (33). While the underlying reason(s) for these findings are not clear, it is possible that the initial (rapid) exercise-induced physical improvements (e.g. gains in muscle strength or function) that participants typically experience during the initial phases of training may contribute to the early improvements in HR-QoL, but as these benefits begin to plateau over time, the changes may become less noticeable, thereby attenuating any perceived improvements in HR-QoL (33). It is therefore possible that the assessment of HR-QoL at baseline and 6 months only in our study may have missed potential short-term improvements in HR-QoL associated with our multi-component exercise training.

There is some evidence that exercise-related improvements in the mental health components of HR-QoL in healthy adults may be related to positive feelings experienced through the social interaction associated with group-based exercise training (35, 37, 38, 39). Although the training program in our study was group-based and run within community leisure centres, there were no positive effects on MCS scores or its subdomains. This may be explained by the exclusion of participants with evidence of depression and cognitive impairment, as previous research has shown that exercise-related impairments in mental health components of HR-QoL are related to lower initial mental HR-QoL scores (39, 40). It is therefore possible that there was a ceiling effect in our study which attenuated any improvement in MCS scores over the 24-week period. This is supported by results from another 24-week RCT conducted in healthy older women with normal baseline HR-QoL scores which reported no improvements in PCS or MCS scores following twice weekly, moderate-to-high intensity resistance training (75–80% RM) (35).

Several strengths of this study include the relatively long intervention period, high mean adherence (∼79%) to the exercise program and the multi-component nature of the exercise program that improved measures of muscle mass, strength and function. Limitations include the inclusion of relatively healthy volunteers who had baseline HR-QoL scores close to the normative values relative to the Australian population which likely blunted the potential to observe any beneficial effects of the intervention and may limit the generalizability of the findings. Furthermore, this study did not include a non-exercising control group and thus it cannot be determined whether either intervention was more effective than usual care. This is important, as is it unknown whether our exercise intervention simply maintained participants' HR-QoL scores over time, thereby explaining the lack of improvement in either group despite improvements in muscle mass, muscle strength and function. Lastly, the singular assessment of HR-QoL throughout the study means that acute changes in HR-QoL attributed to the intervention may have been missed.

In conclusion, this study indicates that a 24-week moderate-intensity multi-component exercise program performed alone or in combination with 160g of cooked lean red meat on each of the three training days per week (a dose in line with current Australian dietary guidelines), was not effective for improving HR-QoL in healthy community-dwelling older adults.

## Trials

This trial registered with the Australian and New Zealand Clinical Trials Registry (ACTRN12613001153707). Data described in the manuscript, code book, and analytic code will not be made available because approval was not sought or granted from the Deakin University Human Research Ethics Committee to freely make available the data.

## Electronic Supplementary Material

Supplementary material, approximately 18.8 KB.
