# Supplementary material for: Effects of a Multi-component, Resistance-Based Exercise Program Combined with Additional Lean Red Meat on Health-Related Quality of Life in Older Adults: Secondary Analysis of a 6-Month Randomized Controlled Trial
Source: J Nutr Health Aging. 2023 Apr 28;27(6):421–9. doi: 10.1007/s12603-023-1915-1 (PMC12880076; doi:10.1007/s12603-023-1915-1)
Supplement: Supplementary file 1 — Supplementary material, approximately 18.8 KB. [file mmc1.docx]

| **Supplementary Table 1:** Mean baseline scores for health-related Quality of Life for the exercise plus lean red meat (Ex+Meat) and exercise plus control (carbohydrate) (Ex+C) group, and the mean within-group changes and mean net differences between the groups for the change after 6 months, for the per protocol analysis. | | | | | | |
| --- | --- | --- | --- | --- | --- | --- |
|  | **Baseline Values and Within-Group Changes** | | | |  | |
|  | **Ex+Meat** | | **Ex+C** | | **Intervention Effects** | |
|  | **Mean ± SD or (95% CI)** | **P-value** | **Mean ± SD or (95% CI)** | **P-value** | **Net Difference (95% CI)** | **P-values**  **Model 1 \| Model 2** |
| **Physical Function** | |  |  |  |  |  |
| Baseline | 47.83 ± 7.00 |  | 49.20 ± 6.47 |  |  |  |
| Δ 6 months | 2.37 (0.90, 3.84) | 0.001 | 1.16 (-0.57, 2.89) | 0.194 | 1.21 (-1.05, 3.47) | 0.276 \| 0.140 |
| **Role Physical** | |  |  |  |  |  |
| Baseline | 48.31 ± 8.51 |  | 50.96 ± 6.74 |  |  |  |
| Δ 6 months | 2.40 (0.33, 4.47) | 0.020 | -0.40 (-2.67, 1.88) | 0.693 | 2.80 (-0.27, 5.86) | 0.066 \| 0.048 |
| **Bodily Pain** | |  |  |  |  |  |
| Baseline | 49.03 ± 10.56 |  | 48.95 ± 9.63 |  |  |  |
| Δ 6 months | -0.71 (-3.12, 1.70) | 0.553 | 0.18 (-2.06, 2.42) | 0.807 | -0.89 (-4.15, 2.36) | 0.549 \| 0.459 |
| **General Health** | |  |  |  |  |  |
| Baseline | 51.66 ± 8.29 |  | 51.25 ± 7.06 |  |  |  |
| Δ 6 months | 0.81 (-0.71, 2.34) | 0.286 | 0.33 (-0.92, 1.58) | 0.562 | 0.48 (-1.46, 2.42) | 0.648 \| 0.557 |
| **Vitality** | |  |  |  |  |  |
| Baseline | 53.25 ± 9.19 |  | 53.43 ± 7.03 |  |  |  |
| Δ 6 months | 0.83 (-1.18, 2.84) | 0.408 | 0.33 (-1.23, 1.90) | 0.626 | 0.49 (-2.00, 2.99) | 0.723 \| 0.641 |
| **Social Functioning** | |  |  |  |  |  |
| Baseline | 51.07 ± 8.97 |  | 52.77 ± 6.51 |  |  |  |
| Δ 6 months | 0.29 (-1.82, 2.40) | 0.784 | -1.78 (-3.81, 0.26) | 0.129 | 2.06 (-0.84, 4.97) | 0.194 \| 0.267 |
| **Role-emotional** | |  |  |  |  |  |
| Baseline | 47.25 ± 10.71 |  | 49.67 ± 9.07 |  |  |  |
| Δ 6 months | 3.12 (0.60, 5.64) | 0.013 | -0.00 (-2.75, 2.75) | 0.788 | 3.12 (-6.83, 0.59) | 0.075 \| 0.080 |
| **Mental Health** | |  |  |  |  |  |
| Baseline | 51.76 ± 8.75 |  | 50.37 ± 7.07 |  |  |  |
| Δ 6 months | -0.61 (-2.66, 1.44) | 0.552 | 1.73 (-0.17, 3.62) | 0.050 | -2.33 (-5.09, 0.42) | 0.080 \| 0.060 |
| **Physical Component Score** | |  |  |  |  |  |
| Baseline | 49.17 ± 7.96 |  | 50.36 ± 7.30 |  |  |  |
| Δ 6 months | 1.38 (-0.29, 3.04) | 0.098 | 0.26 (-1.57, 2.08) | 0.798 | 1.12 (-1.34, 3.58) | 0.356 \| 0.264 |
| **Mental Component Score** | |  |  |  |  |  |
| Baseline | 51.13 ± 9.91 |  | 51.45 ± 7.66 |  |  |  |
| Δ 6 months | 0.63 (-1.53, 2.78) | 0.561 | 0.34 (-1.61, 2.28) | 0.915 | 0.29 (-3.16, 2.58) | 0.714 \| 0.848 |
| **Global Score** | |  |  |  |  |  |
| Baseline | 50.02 ± 6.97 |  | 50.83 ± 5.28 |  |  |  |
| Δ 6 months | 1.06 (-0.23, 2.35) | 0.099 | 0.40 (-0.85, 1.65) | 0.795 | 0.66 (-2.44, 1.16) | 0.340 \| 0.332 |
| All baseline values are unadjusted means ± SD. All change values are unadjusted means (95% CI) and were calculated from the absolute difference from baseline. Mean net differences (95% CI) were calculated by subtracting the within-group changes for the Ex+Meat group from the within-group changes for the Ex+C group after 6 months. Number of participants by group at baseline and 6 months: Ex + Meat, n = 58 and n = 58; C + Ex, n = 64 and n =63. P-values for model 1 were based on an unadjusted model while model 2 included age, sex, number of chronic diseases, change in physical activity, living arrangement and depression/anxiety as covariates. | | | | | | |
